# Supplementary material for: Distinctiveness centrality in social networks
Source: PLoS One. 2020 May 22;15(5):e0233276. doi: 10.1371/journal.pone.0233276 (PMC7244137; doi:10.1371/journal.pone.0233276)
Supplement: S2 File — (PDF) [file pone.0233276.s002.pdf]

Supporting Information

S2 Correlations.

The following tables show the average Spearman's correlation coefficients of distinctiveness centrality with the other centrality indicators considered in the paper, when alpha is equal to 1, 2 or 5. Average correlations were calculated on the 1,000 randomly generated networks, provided as supplementary information files.

In all the tables, we use the following abbreviations: DG = degree; WDG = weighted degree; BTW = betweenness; WBTW = weighted betweenness; CLO = closeness; WCLOS = weighted closeness; EIG = eigenvector centrality; WEIG = weighted eigenvector centrality; CON = constraint; WCON = weighted constraint; ES = effective size; WES = weighted effective size.

Table A1. Average Spearman's correlation coefficients, alpha = 1.

	DG	WDG	BETW	WBETW	CLOS	WCLOS	EIG	WEIG	CON	WCON	ES	WES	D1	D2	D3	D4	D5
**DG**	1.000	0.808	0.907	0.768	0.700	0.525	0.688	0.554	-0.935	-0.876	0.941	0.954	0.829	0.931	0.830	0.780	0.914
**WDG**	0.808	1.000	0.736	0.855	0.595	0.761	0.585	0.739	-0.757	-0.820	0.763	0.773	0.948	0.751	0.954	0.906	0.738
**BETW**	0.907	0.736	1.000	0.759	0.661	0.479	0.577	0.477	-0.940	-0.851	0.946	0.944	0.766	0.865	0.765	0.724	0.853
**WBETW**	0.768	0.855	0.759	1.000	0.550	0.640	0.510	0.580	-0.757	-0.831	0.765	0.775	0.847	0.738	0.862	0.834	0.729
**CLOS**	0.700	0.595	0.661	0.550	1.000	0.646	0.950	0.735	-0.613	-0.602	0.598	0.614	0.469	0.453	0.489	0.424	0.438
**WCLOS**	0.525	0.761	0.479	0.640	0.646	1.000	0.642	0.939	-0.468	-0.545	0.461	0.469	0.594	0.382	0.603	0.529	0.369
**EIG**	0.688	0.585	0.577	0.510	0.950	0.642	1.000	0.760	-0.562	-0.566	0.549	0.571	0.457	0.438	0.478	0.413	0.424
**WEIG**	0.554	0.739	0.477	0.580	0.735	0.939	0.760	1.000	-0.465	-0.538	0.457	0.468	0.564	0.372	0.574	0.499	0.361
**CON**	-0.935	-0.757	-0.940	-0.757	-0.613	-0.468	-0.562	-0.465	1.000	0.895	-0.987	-0.982	-0.800	-0.907	-0.797	-0.754	-0.890
**WCON**	-0.876	-0.820	-0.851	-0.831	-0.602	-0.545	-0.566	-0.538	0.895	1.000	-0.892	-0.899	-0.830	-0.834	-0.832	-0.773	-0.819
**ES**	0.941	0.763	0.946	0.765	0.598	0.461	0.549	0.457	-0.987	-0.892	1.000	0.989	0.815	0.926	0.810	0.770	0.911
**WES**	0.954	0.773	0.944	0.775	0.614	0.469	0.571	0.468	-0.982	-0.899	0.989	1.000	0.821	0.931	0.819	0.778	0.915
**D1**	0.829	0.948	0.766	0.847	0.469	0.594	0.457	0.564	-0.800	-0.830	0.815	0.821	1.000	0.849	0.992	0.971	0.844
**D2**	0.931	0.751	0.865	0.738	0.453	0.382	0.438	0.372	-0.907	-0.834	0.926	0.931	0.849	1.000	0.839	0.813	0.990
**D3**	0.830	0.954	0.765	0.862	0.489	0.603	0.478	0.574	-0.797	-0.832	0.810	0.819	0.992	0.839	1.000	0.980	0.834
**D4**	0.780	0.906	0.724	0.834	0.424	0.529	0.413	0.499	-0.754	-0.773	0.770	0.778	0.971	0.813	0.980	1.000	0.817
**D5**	0.914	0.738	0.853	0.729	0.438	0.369	0.424	0.361	-0.890	-0.819	0.911	0.915	0.844	0.990	0.834	0.817	1.000

Table A2. Standard deviation of Spearman's correlation coefficients, alpha = 1.

	DG	WDG	BETW	WBETW	CLOS	WCLOS	EIG	WEIG	CON	WCON	ES	WES	D1	D2	D3	D4	D5
**DG**	0.000	0.050	0.024	0.064	0.076	0.099	0.075	0.095	0.030	0.039	0.031	0.022	0.041	0.017	0.041	0.049	0.018
**WDG**	0.050	0.000	0.064	0.044	0.089	0.074	0.091	0.083	0.067	0.057	0.067	0.063	0.024	0.068	0.021	0.035	0.068
**BETW**	0.024	0.064	0.000	0.062	0.096	0.112	0.104	0.113	0.018	0.041	0.017	0.017	0.052	0.037	0.053	0.058	0.038
**WBETW**	0.064	0.044	0.062	0.000	0.099	0.100	0.101	0.108	0.065	0.049	0.066	0.061	0.044	0.073	0.040	0.045	0.073
**CLOS**	0.076	0.089	0.096	0.099	0.000	0.077	0.020	0.068	0.102	0.095	0.111	0.103	0.111	0.108	0.106	0.108	0.105
**WCLOS**	0.099	0.074	0.112	0.100	0.077	0.000	0.082	0.024	0.116	0.106	0.120	0.118	0.117	0.126	0.112	0.120	0.121
**EIG**	0.075	0.091	0.104	0.101	0.020	0.082	0.000	0.073	0.109	0.097	0.117	0.108	0.112	0.105	0.106	0.108	0.103
**WEIG**	0.095	0.083	0.113	0.108	0.068	0.024	0.073	0.000	0.120	0.110	0.124	0.121	0.125	0.125	0.118	0.123	0.120
**CON**	0.030	0.067	0.018	0.065	0.102	0.116	0.109	0.120	0.000	0.035	0.012	0.014	0.055	0.038	0.055	0.060	0.041
**WCON**	0.039	0.057	0.041	0.049	0.095	0.106	0.097	0.110	0.035	0.000	0.037	0.033	0.053	0.053	0.053	0.061	0.054
**ES**	0.031	0.067	0.017	0.066	0.111	0.120	0.117	0.124	0.012	0.037	0.000	0.013	0.049	0.030	0.050	0.055	0.032
**WES**	0.022	0.063	0.017	0.061	0.103	0.118	0.108	0.121	0.014	0.033	0.013	0.000	0.045	0.027	0.046	0.050	0.028
**D1**	0.041	0.024	0.052	0.044	0.111	0.117	0.112	0.125	0.055	0.053	0.049	0.045	0.000	0.043	0.003	0.012	0.044
**D2**	0.017	0.068	0.037	0.073	0.108	0.126	0.105	0.125	0.038	0.053	0.030	0.027	0.043	0.000	0.044	0.046	0.005
**D3**	0.041	0.021	0.053	0.040	0.106	0.112	0.106	0.118	0.055	0.053	0.050	0.046	0.003	0.044	0.000	0.008	0.045
**D4**	0.049	0.035	0.058	0.045	0.108	0.120	0.108	0.123	0.060	0.061	0.055	0.050	0.012	0.046	0.008	0.000	0.045
**D5**	0.018	0.068	0.038	0.073	0.105	0.121	0.103	0.120	0.041	0.054	0.032	0.028	0.044	0.005	0.045	0.045	0.000

Table B1. Average Spearman's correlation coefficients, alpha = 2.

	DG	WDG	BETW	WBETW	CLOS	WCLOS	EIG	WEIG	CON	WCON	ES	WES	D1	D2	D3	D4	D5
**DG**	1.000	0.808	0.907	0.768	0.700	0.525	0.688	0.554	-0.935	-0.876	0.941	0.954	0.692	0.758	0.727	0.738	0.895
**WDG**	0.808	1.000	0.736	0.855	0.595	0.761	0.585	0.739	-0.757	-0.820	0.763	0.773	0.617	0.627	0.680	0.897	0.723
**BETW**	0.907	0.736	1.000	0.759	0.661	0.479	0.577	0.477	-0.940	-0.851	0.946	0.944	0.647	0.709	0.685	0.684	0.840
**WBETW**	0.768	0.855	0.759	1.000	0.550	0.640	0.510	0.580	-0.757	-0.831	0.765	0.775	0.621	0.635	0.718	0.821	0.717
**CLOS**	0.700	0.595	0.661	0.550	1.000	0.646	0.950	0.735	-0.613	-0.602	0.598	0.614	0.184	0.196	0.271	0.415	0.427
**WCLOS**	0.525	0.761	0.479	0.640	0.646	1.000	0.642	0.939	-0.468	-0.545	0.461	0.469	0.148	0.228	0.219	0.536	0.359
**EIG**	0.688	0.585	0.577	0.510	0.950	0.642	1.000	0.760	-0.562	-0.566	0.549	0.571	0.171	0.181	0.258	0.404	0.413
**WEIG**	0.554	0.739	0.477	0.580	0.735	0.939	0.760	1.000	-0.465	-0.538	0.457	0.468	0.122	0.184	0.192	0.504	0.352
**CON**	-0.935	-0.757	-0.940	-0.757	-0.613	-0.468	-0.562	-0.465	1.000	0.895	-0.987	-0.982	-0.705	-0.773	-0.727	-0.713	-0.870
**WCON**	-0.876	-0.820	-0.851	-0.831	-0.602	-0.545	-0.566	-0.538	0.895	1.000	-0.892	-0.899	-0.666	-0.706	-0.711	-0.730	-0.802
**ES**	0.941	0.763	0.946	0.765	0.598	0.461	0.549	0.457	-0.987	-0.892	1.000	0.989	0.730	0.802	0.750	0.727	0.893
**WES**	0.954	0.773	0.944	0.775	0.614	0.469	0.571	0.468	-0.982	-0.899	0.989	1.000	0.727	0.793	0.754	0.736	0.896
**D1**	0.692	0.617	0.647	0.621	0.184	0.148	0.171	0.122	-0.705	-0.666	0.730	0.727	1.000	0.902	0.908	0.776	0.841
**D2**	0.758	0.627	0.709	0.635	0.196	0.228	0.181	0.184	-0.773	-0.706	0.802	0.793	0.902	1.000	0.853	0.717	0.925
**D3**	0.727	0.680	0.685	0.718	0.271	0.219	0.258	0.192	-0.727	-0.711	0.750	0.754	0.908	0.853	1.000	0.869	0.841
**D4**	0.738	0.897	0.684	0.821	0.415	0.536	0.404	0.504	-0.713	-0.730	0.727	0.736	0.776	0.717	0.869	1.000	0.760
**D5**	0.895	0.723	0.840	0.717	0.427	0.359	0.413	0.352	-0.870	-0.802	0.893	0.896	0.841	0.925	0.841	0.760	1.000

Table B2. Standard deviation of Spearman's correlation coefficients, alpha = 2.

	DG	WDG	BETW	WBETW	CLOS	WCLOS	EIG	WEIG	CON	WCON	ES	WES	D1	D2	D3	D4	D5
**DG**	0.000	0.050	0.024	0.064	0.076	0.099	0.075	0.095	0.030	0.039	0.031	0.022	0.075	0.065	0.065	0.059	0.021
**WDG**	0.050	0.000	0.064	0.044	0.089	0.074	0.091	0.083	0.067	0.057	0.067	0.063	0.099	0.090	0.087	0.038	0.068
**BETW**	0.024	0.064	0.000	0.062	0.096	0.112	0.104	0.113	0.018	0.041	0.017	0.017	0.084	0.076	0.075	0.065	0.040
**WBETW**	0.064	0.044	0.062	0.000	0.099	0.100	0.101	0.108	0.065	0.049	0.066	0.061	0.096	0.091	0.078	0.049	0.074
**CLOS**	0.076	0.089	0.096	0.099	0.000	0.077	0.020	0.068	0.102	0.095	0.111	0.103	0.134	0.139	0.126	0.109	0.100
**WCLOS**	0.099	0.074	0.112	0.100	0.077	0.000	0.082	0.024	0.116	0.106	0.120	0.118	0.154	0.138	0.146	0.119	0.117
**EIG**	0.075	0.091	0.104	0.101	0.020	0.082	0.000	0.073	0.109	0.097	0.117	0.108	0.131	0.135	0.124	0.109	0.099
**WEIG**	0.095	0.083	0.113	0.108	0.068	0.024	0.073	0.000	0.120	0.110	0.124	0.121	0.155	0.143	0.146	0.122	0.115
**CON**	0.030	0.067	0.018	0.065	0.102	0.116	0.109	0.120	0.000	0.035	0.012	0.014	0.075	0.064	0.070	0.066	0.044
**WCON**	0.039	0.057	0.041	0.049	0.095	0.106	0.097	0.110	0.035	0.000	0.037	0.033	0.082	0.073	0.073	0.066	0.055
**ES**	0.031	0.067	0.017	0.066	0.111	0.120	0.117	0.124	0.012	0.037	0.000	0.013	0.068	0.054	0.063	0.062	0.034
**WES**	0.022	0.063	0.017	0.061	0.103	0.118	0.108	0.121	0.014	0.033	0.013	0.000	0.067	0.056	0.060	0.057	0.030
**D1**	0.075	0.099	0.084	0.096	0.134	0.154	0.131	0.155	0.075	0.082	0.068	0.067	0.000	0.035	0.034	0.061	0.056
**D2**	0.065	0.090	0.076	0.091	0.139	0.138	0.135	0.143	0.064	0.073	0.054	0.056	0.035	0.000	0.047	0.067	0.036
**D3**	0.065	0.087	0.075	0.078	0.126	0.146	0.124	0.146	0.070	0.073	0.063	0.060	0.034	0.047	0.000	0.042	0.049
**D4**	0.059	0.038	0.065	0.049	0.109	0.119	0.109	0.122	0.066	0.066	0.062	0.057	0.061	0.067	0.042	0.000	0.054
**D5**	0.021	0.068	0.040	0.074	0.100	0.117	0.099	0.115	0.044	0.055	0.034	0.030	0.056	0.036	0.049	0.054	0.000

Table C1. Average Spearman's correlation coefficients, alpha = 5.

	DG	WDG	BETW	WBETW	CLOS	WCLOS	EIG	WEIG	CON	WCON	ES	WES	D1	D2	D3	D4	D5
**DG**	1.000	0.808	0.907	0.768	0.700	0.525	0.688	0.554	-0.935	-0.876	0.941	0.954	-0.448	-0.570	-0.687	0.681	0.885
**WDG**	0.808	1.000	0.736	0.855	0.595	0.761	0.585	0.739	-0.757	-0.820	0.763	0.773	-0.681	-0.493	-0.905	0.867	0.714
**BETW**	0.907	0.736	1.000	0.759	0.661	0.479	0.577	0.477	-0.940	-0.851	0.946	0.944	-0.403	-0.500	-0.622	0.630	0.831
**WBETW**	0.768	0.855	0.759	1.000	0.550	0.640	0.510	0.580	-0.757	-0.831	0.765	0.775	-0.549	-0.432	-0.719	0.782	0.708
**CLOS**	0.700	0.595	0.661	0.550	1.000	0.646	0.950	0.735	-0.613	-0.602	0.598	0.614	-0.677	-0.918	-0.610	0.400	0.421
**WCLOS**	0.525	0.761	0.479	0.640	0.646	1.000	0.642	0.939	-0.468	-0.545	0.461	0.469	-0.891	-0.612	-0.843	0.541	0.352
**EIG**	0.688	0.585	0.577	0.510	0.950	0.642	1.000	0.760	-0.562	-0.566	0.549	0.571	-0.678	-0.922	-0.603	0.391	0.409
**WEIG**	0.554	0.739	0.477	0.580	0.735	0.939	0.760	1.000	-0.465	-0.538	0.457	0.468	-0.896	-0.712	-0.825	0.509	0.347
**CON**	-0.935	-0.757	-0.940	-0.757	-0.613	-0.468	-0.562	-0.465	1.000	0.895	-0.987	-0.982	0.370	0.458	0.631	-0.655	-0.859
**WCON**	-0.876	-0.820	-0.851	-0.831	-0.602	-0.545	-0.566	-0.538	0.895	1.000	-0.892	-0.899	0.460	0.468	0.698	-0.664	-0.792
**ES**	0.941	0.763	0.946	0.765	0.598	0.461	0.549	0.457	-0.987	-0.892	1.000	0.989	-0.360	-0.441	-0.631	0.668	0.882
**WES**	0.954	0.773	0.944	0.775	0.614	0.469	0.571	0.468	-0.982	-0.899	0.989	1.000	-0.372	-0.462	-0.638	0.676	0.885
**D1**	-0.448	-0.681	-0.403	-0.549	-0.677	-0.891	-0.678	-0.896	0.370	0.460	-0.360	-0.372	1.000	0.725	0.785	-0.459	-0.199
**D2**	-0.570	-0.493	-0.500	-0.432	-0.918	-0.612	-0.922	-0.712	0.458	0.468	-0.441	-0.462	0.725	1.000	0.560	-0.283	-0.222
**D3**	-0.687	-0.905	-0.622	-0.719	-0.610	-0.843	-0.603	-0.825	0.631	0.698	-0.631	-0.638	0.785	0.560	1.000	-0.677	-0.539
**D4**	0.681	0.867	0.630	0.782	0.400	0.541	0.391	0.509	-0.655	-0.664	0.668	0.676	-0.459	-0.283	-0.677	1.000	0.685
**D5**	0.885	0.714	0.831	0.708	0.421	0.352	0.409	0.347	-0.859	-0.792	0.882	0.885	-0.199	-0.222	-0.539	0.685	1.000

Table C2. Standard deviation of Spearman's correlation coefficients, alpha = 5.

	DG	WDG	BETW	WBETW	CLOS	WCLOS	EIG	WEIG	CON	WCON	ES	WES	D1	D2	D3	D4	D5
**DG**	0.000	0.050	0.024	0.064	0.076	0.099	0.075	0.095	0.030	0.039	0.031	0.022	0.113	0.076	0.076	0.072	0.024
**WDG**	0.050	0.000	0.064	0.044	0.089	0.074	0.091	0.083	0.067	0.057	0.067	0.063	0.086	0.094	0.043	0.045	0.069
**BETW**	0.024	0.064	0.000	0.062	0.096	0.112	0.104	0.113	0.018	0.041	0.017	0.017	0.124	0.102	0.091	0.076	0.042
**WBETW**	0.064	0.044	0.062	0.000	0.099	0.100	0.101	0.108	0.065	0.049	0.066	0.061	0.101	0.103	0.081	0.056	0.076
**CLOS**	0.076	0.089	0.096	0.099	0.000	0.077	0.020	0.068	0.102	0.095	0.111	0.103	0.081	0.033	0.085	0.111	0.098
**WCLOS**	0.099	0.074	0.112	0.100	0.077	0.000	0.082	0.024	0.116	0.106	0.120	0.118	0.041	0.086	0.057	0.116	0.115
**EIG**	0.075	0.091	0.104	0.101	0.020	0.082	0.000	0.073	0.109	0.097	0.117	0.108	0.083	0.032	0.091	0.111	0.097
**WEIG**	0.095	0.083	0.113	0.108	0.068	0.024	0.073	0.000	0.120	0.110	0.124	0.121	0.045	0.076	0.066	0.119	0.113
**CON**	0.030	0.067	0.018	0.065	0.102	0.116	0.109	0.120	0.000	0.035	0.012	0.014	0.124	0.098	0.094	0.075	0.046
**WCON**	0.039	0.057	0.041	0.049	0.095	0.106	0.097	0.110	0.035	0.000	0.037	0.033	0.111	0.094	0.081	0.072	0.056
**ES**	0.031	0.067	0.017	0.066	0.111	0.120	0.117	0.124	0.012	0.037	0.000	0.013	0.130	0.109	0.095	0.072	0.036
**WES**	0.022	0.063	0.017	0.061	0.103	0.118	0.108	0.121	0.014	0.033	0.013	0.000	0.128	0.102	0.093	0.069	0.033
**D1**	0.113	0.086	0.124	0.101	0.081	0.041	0.083	0.045	0.124	0.111	0.130	0.128	0.000	0.072	0.070	0.121	0.129
**D2**	0.076	0.094	0.102	0.103	0.033	0.086	0.032	0.076	0.098	0.094	0.109	0.102	0.072	0.000	0.093	0.113	0.102
**D3**	0.076	0.043	0.091	0.081	0.085	0.057	0.091	0.066	0.094	0.081	0.095	0.093	0.070	0.093	0.000	0.100	0.100
**D4**	0.072	0.045	0.076	0.056	0.111	0.116	0.111	0.119	0.075	0.072	0.072	0.069	0.121	0.113	0.100	0.000	0.065
**D5**	0.024	0.069	0.042	0.076	0.098	0.115	0.097	0.113	0.046	0.056	0.036	0.033	0.129	0.102	0.100	0.065	0.000
